# RETRACTED: Wu et al. Piezoelectric Response of Aligned Electrospun Polyvinylidene Fluoride/Carbon Nanotube Nanofibrous Membranes. *Nanomaterials* 2018, *8*, 420

**DOI:** 10.3390/nano16211433

**Published:** 2026-11-06

**Authors:** Chang-Mou Wu, Min-Hui Chou, Wun-Yuan Zeng

**Affiliations:** Department of Materials Science and Engineering, National Taiwan University of Science and Technology, Taipei 10607, Taiwan; bear200718@gmail.com (M.-H.C.); pinkgirls0111@gmail.com (W.-Y.Z.)

The journal retracts the article titled “Piezoelectric Response of Aligned Electrospun Polyvinylidene Fluoride/Carbon Nanotube Nanofibrous Membranes” [1], as cited above.

Following the publication, concerns were brought to the attention of the editorial office regarding the integrity of several images presented in this paper [1].

In accordance with standard journal procedures, the editorial office and editorial board conducted an investigation that identified indications of inappropriate image editing and overlap of panels within Figure 1, and between Figures 2 and 4. Although the authors collaborated with the investigation, they were unable to provide a satisfactory explanation for the concerns identified or the underlying data, and they subsequently requested that the article be retracted. As a result, the editorial board has decided to retract this article in accordance with MDPI’s retraction policy.

This retraction was approved by the Editor-in-Chief of the journal *Nanomaterials*.

The authors have been informed of this retraction but did not provide a comment on this decision.
